# New Approaches in Drug Dependence: Opioids

**DOI:** 10.1007/s40429-021-00373-9

**Published:** 2021-05-26

**Authors:** Juliane Mielau, Marc Vogel, Stefan Gutwinski, Inge Mick

**Affiliations:** 1 Department of Psychiatry and Psychotherapy, Psychiatric Hospital of Charité at St. Hedwig Hospital, Große Hamburger Straße 5- 11, 10115 Berlin, Germany; 2 Department of Addictive Disorders, Psychiatric University Clinic Basel, Basel, Switzerland; 3 Department of Addictive Disorders, Psychiatric Services Thurgau, Muensterlingen, Switzerland

**Keywords:** Opiod substitution, Heroin-assisted treatment, Harm reduction

## Abstract

**Purpose of Review:**

This article aims to provide an overview of standard and adjunctive treatment options in opioid dependence in consideration of therapy-refractory courses. The relevance of oral opioid substitution treatment (OST) and measures of harm reduction as well as heroin-assisted therapies are discussed alongside non-pharmacological approaches.

**Recent Findings:**

Currently, recommendation can be given for OST with methadone, buprenorphine, slow-release oral morphine (SROM), and levomethadone. Heroin-assisted treatment using diamorphine shall be considered as a cost-effective alternative for individuals not responding to the afore-mentioned opioid agonists in order to increase retention and reduce illicit opioid use. The modalities of application and the additional benefits of long-acting formulations of buprenorphine should be sufficiently transferred to clinicians and the eligible patients; simultaneously methods to improve planning of actions and self- management need to be refined. Regarding common primary outcomes in research on opioid treatment, evidence of the effectiveness of adjunctive psychological interventions is scarce.

**Summary:**

Maintaining a harm reduction approach in the treatment of opioid addiction, a larger range of formulations is available for the prescribers. Embedding the pharmacological, ideally individualized treatment into a holistic, structure-giving concept also requires a reduction of fragmentation of ancillary services available, drug policies, and treatment philosophies on a global scale.

The United Nations Office on Drugs and Crime (UNODC) reported a worldwide increase of drug usage of 30% in 2018, compared with 2009, stating that in 2019 thirty-five million people fulfill the criteria of drug use disorders [1]. The National Center of Health Statistics observed a 10% increase of overdose deaths involving synthetic opioids other than methadone in the USA in 2018, compared to the previous year [2]. During the last decades, controversial public debates on the most effective treatment of addiction as a chronically relapsing illness have been held [3]. An estimation of the economic burden of prescription, opioid overdose, abuse, and dependence in the USA amounted to $78.5 billion in 2013 [4]. An executive summary published in 2017 by the Council of Economic Advisers denoted that prior publications have underestimated the cost of the so-called opioid crisis in the USA, stating an economic burden six times higher than previous estimates [5]. Fatalities (85% of total cost) and healthcare expenses, as well as foregone earnings from employment and higher costs to the criminal justice system, are relevant in this context [5]. Additionally, a disruption of drug trafficking due to the COVID-19 pandemic favored diminished purity of illicit drugs and lead to rising prices for cocaine and heroin [6]. Under given circumstances and global developments, it seems highly relevant to address a growing proportion of patients who do not benefit sufficiently from standard treatments of opioid dependence. Several international guidelines, for instance, submitted by the World Health Organization or the German Medical Association, recommend OST with methadone (methadone maintenance treatment: MMT), levomethadone, buprenorphine (BUP), or slow-release morphine (SROM) for opioid dependence [7, 8]. Methadone is prescribed in primary care clinics in Great Britain, Canada, and Australia, but it is not approved for the treatment of OUD in primary care settings in the USA [9].

Methadone treatment decreased the mortality rate when comparing street heroin addicts with patients in MMT (63 versus 8 times increased mortality) and an official statistics group and limits the burden of disease due to a reduction of infectious diseases [10, 11]. Methadone (D-, L-methadone) and levomethadone (isolated L-methadone), acting as full agonists at μ-receptors, entail a larger risk of fatal overdose due to respiratory depression and can lead to long-lasting withdrawal symptoms in detoxification compared to BUP [12]. Daily dosing is needed, and at times, take-home medication may be diverted to other illicit drug users [13]. Other experienced side effects in MMT are constipation, sweating, dry mouth, malaise, joint pain, reduced sexual desire, or a decreased ability to orgasm [14, 15]. The tolerability of methadone can be limited in patients with prolongation of the electrocardiographic QTc interval [16, 17], comedication of inhibitors/inducers of the cytochrome P450 enzymes [18, 19], severe hepatic insufficiency [18], or rapid metabolizers [20, 21]. The d-isomer does not bind to μ-receptors but demonstrates an effect on NMDA receptors that conceivably reduces the development of tolerance [22, 23]. Consequently, patients on levomethadone may need higher equivalent dosages than those individuals on methadone [24]. However, in long-term MMT (mean treatment duration of 7.5 years), Gutwinski et al. did not confirm a significant difference between racemic methadone and levomethadone regarding the development of tolerance [25].

## Harm Reduction Programs

Harm reduction subsumes a variety of compassionate and pragmatic strategies focusing on minimizing substance-related harm and enhancing quality of life for affected individuals and their communities without requiring abstinence or reduction of consumption quantities [26]. The effectiveness of improved policies and new programs was scientifically evaluated alongside ethical research in users of illicit substances [27]. The development of lower-threshold, patient-centered interventions seemed especially auspicious for the multi-morbid and high-utilizing population of drug users. The harm reduction approach allows for a broad range of treatment results ranging from survival to full recovery and does not only play a major part in OST but also in dependence on other legal and illicit substances. The overall quality of evidence on the efficacy of electronic nicotine delivery systems (ENDS) analyzed by Hartmann-Boyce et al. in 2016 [28] was graded as low (to very low), so that national guidelines do not state a recommendation for ENDS as a means of harm reduction in nicotine dependency. In alcohol-dependent homeless individuals, a monthly injected extended-release naltrexone formulation and harm reduction counseling were regarded as promising means of supporting reductions in alcohol use and alcohol-related harm [29]. In reaction to an increase of opioid-related overdose deaths, North Carolina legalized syringe exchange programs in 2016 and distributed naloxone, a non-addictive medication, which reverses opioid overdose, into the community [30]. This may underline the importance of the harm reduction debate for opioid dependence treatment, although this article does not intend to give a complete review on this subject.

## Differences Between the Established Forms of OST

Advantages of the partial opioid receptor agonist BUP are less severe withdrawal symptoms, a diminished risk of overdosing, and a longer duration of action allowing alternate day dosing [31]. Common side effects in BUP substitution therapy are anxiety, sleep disorders, constipation, and headache [7]. A “clear state of consciousness” is described as a particularity of BUP treatment [32] that underlines its suitability for individuals with stable living conditions, high motivation, and low psychiatric comorbidity [33, 34]. A Cochrane review of data from randomized controlled trials summarized that it was statistically superior to placebo in retaining heroin users in OST and was effective in suppressing heroin use when a daily dose of at least 8mg BUP was chosen [35]. When compared with methadone, however, BUP prescribed in flexible doses was inferior in terms of retaining patients in treatment [35]. A British study by Pinto and colleagues that explored patient preferences (BUP or MMT) included 361 opioid-dependent individuals, most of which (63%) chose methadone over BUP [36]. On a side note, selection of methadone was associated with more severe substance use problems and psychological strain. It was demonstrated that 6-month retention rates were higher with methadone versus BUP (69.6% versus 42.5%, *p* < .001), although fewer opioid-positive urine specimens were registered in the BUP group and a smaller risk of death during induction was evident [37]. An evaluation of acceptance of prescribed intravenous BUP has been done in France where diacetylmorphine programs face political or regulatory difficulties. Eighty-three percent of 371 participants would be interested in the afore mentioned substance. Additionally, a larger amount of BUP receivers, as compared to heroin or morphine sulfate injectors with a greater number of complications, were more prone to accept treatment with intravenous BUP [38]. One should be aware that despite a proven reduction of positive subjective effects of other opioids, its abuse liability, when administered intravenously or intranasally, remains evident in patients treated with a low dose of sublingual BUP [39]. In a Finnish sample (*N* = 1508) being treated with buprenorphine-naloxone (BNX) (OR 2.60, *p* = 0.005) in a low dosage (< 9.0 mg/day; OR 5.70, *p* < 0.001) and being consulted in a healthcare center (OR 2.03, *p* = 0.029) were factors associated with the injection of a patient’s own OST medication [40]. Injection of illicit OST medications was found more frequently in low-dose BNX treatment and when insufficient psychosocial support or additional use of psychotropic medications from illicit markets was evident.

A mu-opioid receptor agonist SROM guarantees steady blood levels over 24 h [41, 42] and can serve as an alternative substitution treatment [43] for patients responding poorly to methadone [44] and other substances available for OST or those individuals suffering from intolerable side effects. Having long-standing experience of prescription of slow-release morphine (SROM) in OST, Austrian clinicians reported in a cohort analysis of 5165 participants that the retention rate after 1 year of treatment with SROM amounted to 79 % (in comparison retention rate for methadone treatment 59%) [45]. An RCT from 2014 compared SROM and methadone in terms of illicit heroin use and concomitant drug consumption [46]. Non-inferiority of SROM treatment was found in a sample of 157 participants whereby a dose effect became evident for SROM substitution therapy (decreasing proportions of heroin-positive urine samples with increasing doses). In an international, multi-center, two-phase study, safety and efficacy of SROM versus methadone were investigated (198 participants entered phase two) [47]. The authors report that patients showed significantly less prolongation of QTc intervals under treatment with SROM, and reported higher treatment satisfaction, fewer cravings for heroin, as well as lower mental stress levels. A recent non-interventional naturalistic observational study provides first data on the switching process of BUP to SROM [48] which was completed for more than 75% of participants from one day to the next. Since 34% of substituted patients receive BUP in the European Union [49], further studies shall monitor the willingness and, in some cases, necessity to opt for SROM treatment.

## Non-pharmacological Strategies and Setting Variables

Regarding the financial strain on public health systems caused by relapse and the subsequently offered inpatient treatments, an improvement of concurrent non-pharmacological strategies and adaptation of setting variables in OST should be discussed. This may be instrumental in reducing drop-outs from long-term maintenance treatments. In an RCT by Fiellin et al. (2006), it was investigated whether adding counseling to BNX treatment impacts on the self-reported frequency of illicit opioid use, the percentage of opioid-negative urine specimens, and the maximum number of consecutive weeks of abstinence from illicit opioids. Among 166 patients receiving BNX in primary care for opioid dependence, the efficacy of brief weekly counseling and once-weekly medication dispensing did not differ significantly from that of extended weekly counseling and thrice-weekly dispensing [50]. A multi-site RCT by Weiss et al. used a two-phase adaptive treatment design to compare the efficacy of BNX as part of a brief versus extended treatment program and differing intensities of adjunctive counseling (standard medical management (SMM), standard medical management plus individual opioid dependence counseling (OPC)) [51]. The brief treatment consisted of a 2-week stabilization on BNX, a 2-week taper, and 8-week postmedication follow-up. The second phase (extended treatment) comprised a 12-week stabilization, a 4-week taper, and 8-week postmedication follow-up. Only 6,6% of 653 participants successfully completed the first phase and therefore exited the program, irrespective of whether SMM or SMM plus OPC was received. In week 12 of phase 2 treatment, 49,3% of participants maintained successful outcomes (defined as abstaining from opioids during the last week of stabilization and at least for 2 weeks between weeks 9 and 11). Nevertheless, a decline of the success rate to 8,6% with no counseling difference was observed at the end of the second phase. In 2012, Miotto et al. investigated the role of treatment setting in buprenorphine treatment programs comparing individual counseling, group counseling utilizing the manualized matrix model of cognitive-behavioral treatment, and a private clinic setting reflecting standard medical management [52]. While the authors concluded that treatment with BUP is feasible in various treatment settings, the retention differed by treatment site – group counseling programs showed significant therapeutic success, while a private clinic setting did not. Interestingly, differences in staff attitudes between national OST centers in Norway have also been found to be associated with measurable differences in caseload, intensity of case management, and patient outcomes [53]. “Rehabilitation-oriented” centers were characterized by smaller caseloads, more frequent urine drug testing, intensified case management, and had less drug use among their patients. Nevertheless, “intermediate” centers had the lowest treatment termination rate. However, despite vast literature on this subject, a final statement on the optimal treatment remains a challenge. For example, although it is widely assumed that psychological interventions are an essential part of drug dependence treatment, a recent Cochrane review of psychosocial elements as an adjunct to methadone treatment found that such interventions failed to improve outcomes in terms of retention, non-prescribed opioid use, psychiatric symptoms, compliance, or depression [54]. A review released in 2017 thematized the effectiveness of supervised dosing as compared with dispensed take-home medication and led to the inclusion of six studies (*N* = 7999). No evidence could be provided after *3 months and thereafter* in terms of benefit of the supervised dosing with respect to retention in treatment, reduction of opioid use, decreased mortality, and adverse drug events [55]. A reduction of diversion was reported in supervised dosing by the study of Holland et al. [56]. Primary barriers to OST, such as waiting lists, strict rules regarding abstinence, limited take-home dose availability, and lack of information on treatment options, were highlighted in a German questionnaire-based study that elucidated the mismatch of patients’ requests for treatment and the amount of physicians actively providing OST [57]. A variety of setting factors associated with higher OST program effectiveness and retention have been identified: flexible clinic policy (i.e., an orientation to maintenance as opposed to lower doses and abstinence) [58, 59], optimized counseling [59, 60], less expensive treatment fees [59, 60], and greater accessibility [61].

## Heroin-Assisted Treatment

Since the 1990s, studies have investigated alternatives to standard OSTs as demonstrated in 1994 in Switzerland, offering injectable heroin to non-responders to OST [62]. Haasen et al. substantiated the positive effects of heroin-assisted treatment (HAT) highlighted in uncontrolled [63] and controlled [64, 65] trials for persons resistant to methadone treatment [66]. Methadone as the most frequently provided opioid pharmacotherapy in MT is nowadays available in diverse modalities but fails to reach a considerable number of illicit opiate users [67]. Furthermore, Bald et al. showed that a large number of patients in conventional OST would prefer HAT, in particular participants on higher dosages of methadone, with more than five detoxifications and continued illicit drug use [68]. Treatment study reviews of the 1990s pointed out that 30–70% of subjects leave methadone treatment within the first 2 years [69, 70]. More recently analyzed data from a national opioid substitution case register (1992-2012) suggested that patients receiving OST tend to alternate between exiting and restarting OST therapy [71, 72].

A review by Ferri et al. (2011), including eight RCT’s, has referred to secondary outcomes of HAT such as criminal activity, integration at work, and family relationships [73]. Regarding work integration, Haasen et al. reported an improved employment status among study participants, from 4.4% at baseline to 10.6% at month 12, with heroin group participants doing slightly better than methadone participants [66]. The NAOMI study also described an improvement of employment satisfaction and social relations in the heroin groups [74]. Comparisons between the HAT and methadone or other opioid agonist treatments for opioid dependence with regard to family relationships did not confirm significant differences whereby this secondary outcome was only addressed in 4 studies. Several studies have shown the effectiveness of injectable diamorphine in terms of increased retention and reduced illicit opioid use [74–77]. Diamorphine Diamorphine hydrochloride is *administered* in the UK, Switzerland, Germany, the Netherlands, Luxemburg, Canada, and Denmark and is known for reduced criminality and improved physical, mental, and social health [78]. In Belgium and Spain, application of diamorphine has been legitimized for certain research settings. This type of treatment however requires up to three doses per day [79] and is used in circa 5 to 8% of all OATs in the afore-listed countries [75]. Preparations of diacethylmorphine in tablet form or intranasal are currently used in Switzerland as part of an individualized therapy in defined indications [80].

Trials reporting instances of sudden-onset respiratory depression in outpatients receiving injectable diamorphine constituted a rate of circa 1 in 6000 injections that can be successfully managed in highly structured and supervised treatment programs (74; 80). Bell *et al. díscussed* two main reasons for superiority of diamorphine in case of failed methadone treatment: less side effects or greater control of withdrawal symptoms and motivational effect through access to a highly selective form of treatment [81]. A fraction of methadone “non-responders” that were prospectively qualifying for diamorphine complied with 6 months of intravenous heroin treatment and responded [64]. Participating individuals attended three times per day, were introduced to safe i.v. injection practices, and observed for 30 min after self-injection. Psychological counseling, counseling on prevention of HIV, social as well as legal assistance and basic somatic care were available to all participants. This finding suggests that the prospect of a potent second-line treatment acts as an incentive to MT. Moreover, diamorphine is evaluated as a transitional step in social reintegration [82] and a supplement for MMT rather than its substitute. A cost-benefit analysis study in Germany reported that HAT produced a net savings balance (€5,966) per patient per year, whereas the costs of OST remained greater than its calculated savings (minus €2,069) because of its insufficient reduction of crime and criminal justice system costs [83]. A similar result has been demonstrated in a Dutch analysis in 2005 [84].

## Injectable Hydromorphone Hydrochloride

In 2016, a non-inferiority trial compared injectable hydromorphone hydrochloride and diacetylmorphine regarding the reduction of illicit heroin use in a sample of 202 chronic i.v. opioid users after 6 months of intervention [85]. Proclaiming a consistency of the primary outcome (number of self-reported days of street heroin use in the prior 30 days) with prior diamorphine trials, a non-inferiority of hydromorphone was shown in per-protocol analysis. With regard to the co-primary outcomes (number of days of using any street-acquired opioids in the prior 30 days, proportion of urinalyses positive for street heroin markers at the 6-month assessment), hydromorphone was not inferior in intention-to-treat and per-protocol-analyses. In settings where diamorphine is not legally available for OST, hydromorphone as a substance readily applied in pain therapy could enlarge the scope of treatment for those individuals not responding to oral OST or buprenorphine. To date, the NAOMI study was the only work testing hydromorphone against oral methadone treatment whereby just 10% of participants (*N* = 25) were randomized to the HDM group [74]. Within the intravenously treated groups, participants could not achieve an accurate differentiation between diacetylmorphine and hydromorphone. Observation of similar outcomes for these two drugs shall be regarded with a considerable lack of power, but authors already envisioned a potential gain of HDM due to diminished regulatory obstacles. Moreover, a within-trial analysis by Bansback et al. confirmed that HDM treatment offered in the SALOME study produced similar quality-adjusted life years results as compared with diamorphine at slightly higher costs [85]. Modeling outcomes during a patient’s lifetime insinuated that i.v. HDM might prove greater benefit than methadone and be cost-cutting thanks to decreased criminal involvement [86].

## Newly Developed Long-Acting Formulations

Another approach to improve treatment outcomes in terms of abstinence from illicit drugs is subcutaneous application of BUP depots. Strengths of this application form are seen in an improved medication adherence due to avoidance of peaks and troughs as well as reduced stigmatization (non-daily application facilitates social and occupational integration). Additionally, diversion of BUP and intravenous misuse of the prescribed medication is impossible [87]. Lofwall et al. concluded in their RCT from 2018 that depot BUP, administered either weekly or monthly, did not result in an inferior likelihood of being a responder or having urine test results negative for opioids compared to sublingual BNX [88]. A 6-month BUP implant was given to 84 opiate dependents in an intention-to-treat population and compared with 78 patients of a sublingual BUP group [89]. Responder’s proportion, defined as more than 4 of 6 months without illicit opioid use, was 96.4% in the BUP implants group versus 87.6% in the sublingual BUP group, demonstrating statistical non-inferiority. It shall be emphasized that the included patients were a priori stable opiate dependents on a rather low daily sublingual BUP dose of 8 mg (or less) per day and therefore being hardly representative of the majority of opioid substitution seeking patients. Long-acting formulations of BUP that were FDA-approved for treatment of OUD in the USA between 2016 and 2018 are RBP-6000 (Sublocade®), CAM2038 (Brixadi® or Buvidal® in Europe/Australia), and Probuphine (Sixmo® in EU) [90]. As recently thoroughly discussed by Ling et al., the additional value of these formulations is highly dependent on the readiness of clinicians to provide “procedures” for injectable or implantable BUP. Furthermore, a dogma being uphold by a fraction of the addiction aid system that declares detoxification to be the ultimate path of recovery should be mitigated to allow a greater acceptance of long-term recovery among stakeholders [90]. The liberty gained due to infrequent applications of BUP creates the challenging task of finding structural elements in daily life, which are not determined by the procurement of illicit substances.

## Recent and Experimental Approaches

Driven by findings of epidemiological studies, authors focused on the adverse and beneficial effects of medical cannabinoids on opioid sensitivity during the last 10 years. Cannabidiol has especially raised interest, since an associated reduction of the reward-facilitating effect of morphine [91] and cue-induced heroin-seeking behavior has been affirmed [92]. The non-rewarding cannabinoid has shown to provoke diminished cue-induced cravings and a reduction of anxiety in individuals who are abstinent from heroin use in clinical pilot studies [93]. The wide safety margin and the protracted action of cannabidiol underline its potential relevance for adjunctive treatment in opioid disorders, although its use at present is not being specifically defined for opioid dependents [94–96].

Finally, studies on pharmacogenomics pose the question, whether functional differences of OPRM1 gene variants could advance the improvement of the pharmacological response in opioid dependence treatment [97]. Apart from a predictive value for opioid dose in OST, prevention of adverse effects and identification of drug-naive individuals being at elevated risk of addiction when treated with opioid-based analgesics might be facilitated by future pharmacogenetic recommendations [97].

## Conclusions

Being premised on harm reduction, the effectiveness of OST is indisputable. The introduction of different forms of application and types of opioids allows to integrate individual needs of patients. Innovative pharmacological measures in pain therapy concerning forms of application or approaches (gene therapy) shall be continuously evaluated for a possible transfer to treatment of opiate dependence. The risk to cause addiction, particularly when opiates are used, needs particularly thorough evaluation in pain management of persons with diagnosed addictions. Moreover, screening of psychiatric comorbidities should sensitize professional to address syndromes not only with pharmacological measures but also consider psychotherapeutic elements concerning comorbidities, such as posttraumatic syndromes and personality disorders. Diamorphine programs in Germany are, for instance, mostly based on a holistic and multi-professional concept offering a quasi day clinic character which enables to meet the needs of psychiatric comorbidities such as posttraumatic disorders.
